# A new species of *Pseudomegischus* van Achterberg from China, with a key to the species (Hymenoptera, Stephanidae)

**DOI:** 10.3897/zookeys.537.6592

**Published:** 2015-11-18

**Authors:** Jiang-Li Tan, Xu-Lei Fan, Cornelis van Achterberg, Tao Li

**Affiliations:** 1 Key Laboratory of Resource Biology and Biotechnology in Western China (Northwest University), Ministry of Education; School of Life Sciences, Northwest University, 229 North Taibai Road, Xi’an, Shaanxi 710069, China; 2 General Station of Forest Pest Management, State Forestry Administration, Shenyang 110034, China

**Keywords:** *Pseudomegischus*, new species, new record, southern China, Jiangxi, key

## Abstract

The genus *Pseudomegischus* van Achterberg, 2002, is newly reported from China. A new species, *Pseudomegischus notiochinensis* **sp. n.**, is described and illustrated from southern China. A key to the species of *Pseudomegischus* is included.

## Introduction

The small genus *Pseudomegischus* van Achterberg, 2002 (Hymenoptera: Stephanidae) has an Indo-Australian distribution and contains four described species. The species were revised by van Achterberg (2002) and the nearest known locality of the genus (compared to China) is the southern Philippine island of Mindanao. Recently, one of us (TL) reared a series obviously belonging to a new species of *Pseudomegischus*. It is the first host record of a species of this genus and the first species known from the Asian continent. There are several host records of Stephanidae, mainly of the genus *Foenatopus* Smith, 1860 (Aguiar 2004, Aguiar et al. 2010) and mainly concerning Buprestidae and Cerambycidae (Coleoptera). In China Stephanidae has been reported from Buprestidae by Chao (1964) (*Megischus ptosimae* Chao, 1964, reared from *Ptosima chinensis* Marseul, 1867 in peach trees) and Tan et al. (2015) (*Schlettererius determinatoris* Madl, 1991, reared from *Chrysobothris succedana* (Saunders) in *Larix* sp.). Van Achterberg and Yang (2004) reported ovipositing in Buprestid and Cerambycid larvae in various trees by *Megischus tridentatus* van Achterberg & Yang, 2004. The only case of biological control involving Stephanidae is the Nearctic *Schlettererius cinctipes* (Cresson, 1880) introduced to Tasmania from California for control of the introduced *Sirex noctilio* (Fabricius, 1793) (Hymenoptera: Siricidae; Taylor 1967; van Achterberg 2002).

## Material and methods

The specimens studied of *Pseudomegischus* spp. belong to the collection of the Insect Museum of the General Station of Forest Pest Management, Shenyang (GSFPM), P.R. China, some paratypes are deposited in the insect collection of the Northwest University, Xi’an (NWUX), P.R. China, and the Naturalis Biodiversity Center, Leiden (RMNH), the Netherlands.

The morphological terminology follows van Achterberg (2002) and a key to the genera is present in this paper and in Hong et al. (2011). Observations and descriptions were made with an Olympus SZX11 stereomicroscope and fluorescent lamps. Photographic images were made with the Keyence VHX-5000 digital microscope and processed with Adobe Photoshop CS5.

## Taxonomy

### Pseudomegischus

Taxon classificationAnimaliaHymenopteraStephanidae

van Achterberg, 2002

Figs 1–3 , 4–13

Pseudomegischus van Achterberg, 2002: 169; Aguiar 2004: 73–74 (list of literature); Hong et al. 2011: 7. Type species (by original designation): *Stephanus sulcifrons* Schletterer, 1889.

#### Diagnosis.

**Figures 1–3.**
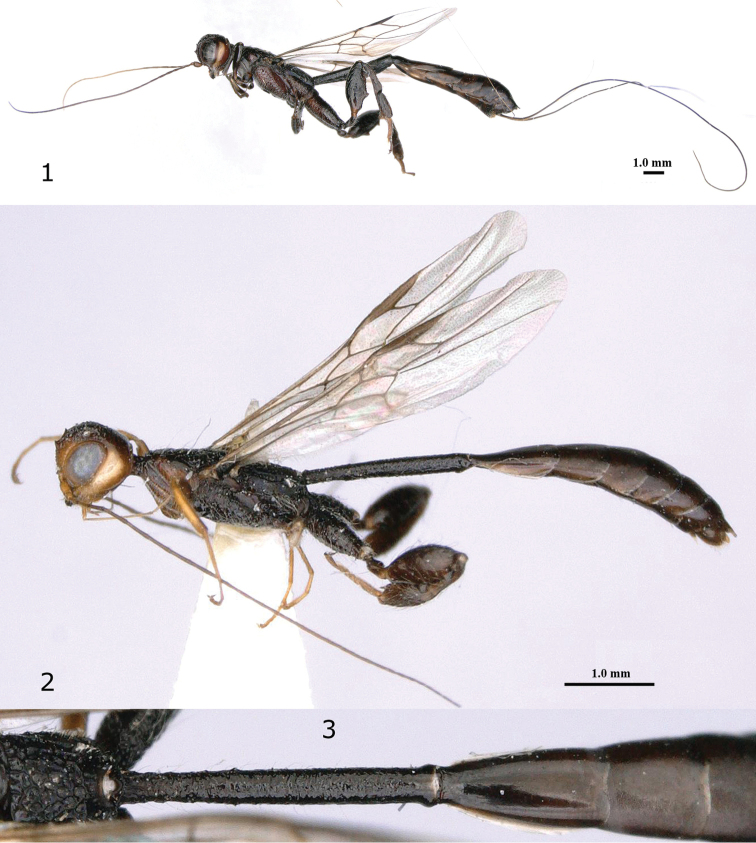
*Pseudomegischus notiochinensis* Tan & van Achterberg, sp. n. **1** holotype, female, habitus lateral **2** paratype, male, habitus lateral **3** first metasomal tergite (T1) of male dorsal.

**Figures 4–13.**
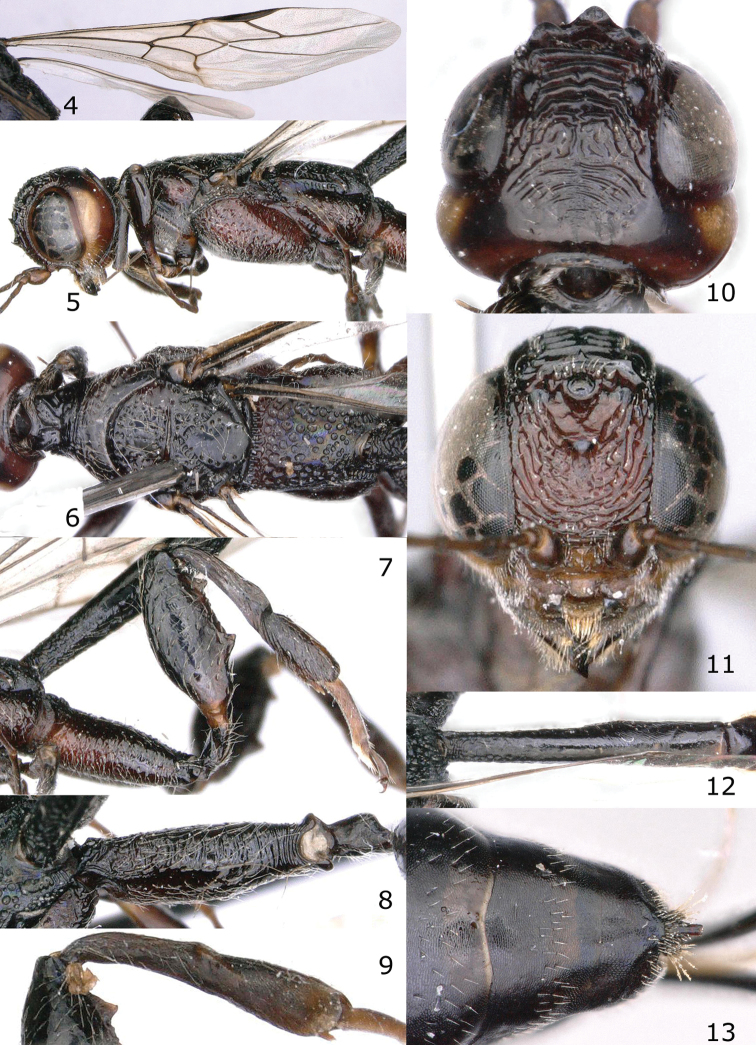
*Pseudomegischus notiochinensis* Tan & van Achterberg, sp. n., female, holotype. **4** wings **5** mesosoma lateral **6** mesosoma dorsal **7** hind leg lateral **8** hind coxa dorsal **9** hind tibia inner side **10** head dorsal **11** head anterior **12** first metasomal tergite dorsal **13** apical metasomal segments, showing pygidial process.

Temple with pale yellowish streak (Fig. 5); vertex anteriorly and stemmaticum (= ocellar area) with shallow median groove (Figs 10–11); pronotum with weak or strong transverse protuberance (Fig. 3); neck with two strong converging carinae laterally and antero-medially with triangular protuberance (Fig. 6); vein 1-M of fore wing 3.1–4.8 × vein 1-SR; vein 1-SR of fore wing straight (Fig. 4); hind tibia with small round pit at top of depression; outer side of hind tibia with oblique striae or carinae (Fig. 7); hind femur with two large teeth (Fig. 7); pygidial process in both sexes present (Fig. 13); ovipositor sheath without ivory subapical band (Fig. 1).

#### Distribution.

Indo-Australian.

#### Biology.

Presumably ectoparasitoid of Cerambycidae and/or Siricidae.

#### Key to species of the genus *Pseudomegischus*

**Table:** 

1	Hind basitarsus ivory or pale yellowish or light brown, distinctly contrasting with dark brown middle of hind tibia (fig. 603 in van Achterberg 2002); hind tibia with some fine oblique striae dorsally	**2**
–	Hind basitarsus yellowish-brown to dark reddish-brown, less contrasting with blackish middle of hind tibia (Fig. 7; figs 255, 265 in van Achterberg 2002); hind tibia mainly smooth, at most with some obsolescent striae dorsally (Fig. 9)	**3**
2	First submarginal cell of fore wing ends near level of apex of pterostigma; head infuscate dorsally; pale yellowish streak behind eye distinct (fig. 607 in van Achterberg 2002); vein 2-SR of fore wing about 1.2 × as long as vein r; median carina of neck distinct (fig. 609 l.c.); mesopleuron only antero-medially coarsely reticulate, remainder largely smooth; Philippines	***Pseudomegischus rugipleurae* (Elliott, 1928)**
–	First submarginal cell of fore wing ends distinctly beyond level of apex of pterostigma (fig. 604 in van Achterberg 2002); head dark yellowish-brown dorsally; pale yellowish streak behind eye indistinct (fig. 600 l.c.); vein 2-SR of fore wing about 1.4 × as long as vein r (fig. 604 l.c.); median carina of neck largely absent (fig. 601 l.c.); mesopleuron medially coarsely remotely punctate; Indonesia (Mysol)	***Pseudomegischus insidiator* (Smith, 1863)**
3	Vein 1-M of fore wing 4.8–5.4 × as long as vein 1-SR (fig. 261 in van Achterberg 2002); hind femur with distinct third medium-sized tooth behind large apical tooth (fig. 265 l.c.); hind basitarsus more or less infuscate dark reddish-brown, dark brown or largely blackish-brown, not distinctly contrasting with blackish hind tibia (fig. 265 l.c.); pale streak of temple rather differentiated (fig. 261 l.c.); length of ovipositor sheath about 2.1 × fore wing; Philippines, Malaysia (Sabah)	***Pseudomegischus sulcifrons* (Schletterer, 1889)**
–	Vein 1-M of fore wing about 3 × as long as vein 1-SR (Fig. 4; fig. 257 in van Achterberg 2002); hind femur without distinct third medium-sized tooth behind large apical tooth, at most with undefined protuberance (Fig. 7; fig. 254 l.c.); hind basitarsus orange-brown, rather contrasting with blackish hind tibia (fig. 254 l.c., but less so in *Pseudomegischus notiochinensis* (Fig. 7) or basitarsus and tibia similarly coloured); pale streak of temple not well differentiated (fig. 252 l.c., but distinct in *Pseudomegischus notiochinensis*; Fig. 5); length of ovipositor sheath about 2.3 × fore wing	**4**
4	Head pale yellowish brown and without distinctly differentiated ivory streak of temple but dorsally and anteriorly darkened (figs 252–253 in van Achterberg 2002); propodeum medio-dorsally coarsely rugose (fig. 256 l.c.); fore wing membrane evenly brownish, but becoming paler apically (fig. 257 l.c.); mesopleuron coarsely punctate, with interspaces at most as wide as punctures; hind femur moderately widened medially in lateral view (fig. 254 l.c.); Indonesia (Sulawesi)	***Pseudomegischus celebensis* van Achterberg, 2002**
–	Head dark brown except for distinct ivory streak of temple (Fig. 10); propodeum medio-dorsally regularly scrobiculate (Fig. 6); fore wing membrane largely subhyaline or slightly brownish (Fig. 4); mesopleuron moderately punctate, with smooth interspaces much wider than punctures (Fig. 5); hind femur strongly widened medially in lateral view (Fig. 7); China (Jiangxi)	***Pseudomegischus notiochinensis* sp. n.**

### Pseudomegischus notiochinensis

Taxon classificationAnimaliaHymenopteraStephanidae

Tan & van Achterberg sp. n.

http://zoobank.org/E51424F8-202F-49B6-B4EC-C8659C8E3AF8

Figures 1–3 , 4–13

#### Type material.

Holotype, ♀ (GSFPM): “**China**: Jiangxi, Quannan, 8.v.2009, Shichang Li, [reared from branches of] *Castanopsis kawakamii* Hay.”. Paratypes: 3♀8♂ (NWUX, RMNH, GSFPM): same data except collecting date 4, 10, 12 or 18.v.2009, and from *Castanopsis kawakamii* Hay. or *Castanopsis faberi* Hance.

#### Diagnosis.

Head in dorsal view parallel-sided behind eyes (Fig. 10); posterior half of pronotum comparatively low and dorso-posteriorly finely transversely rugose (Fig. 6); first subdiscal cell of fore wing comparatively robust and 2.5–2.9 × longer than wide (Fig. 4); hind coxa with strong and sparse rugae, and without dorsal tooth (Fig. 8); first-third metasomal tergites black or dark brown (Fig. 1); first metasomal segment narrow in lateral view (Figs 1, 2); first tergite 6.0–7.6 × (♀♂) as long as its maximum width and irregularly coarsely transversely rugose (Figs 3, 12).

The new species runs to *Pseudomegischus celebensis* van Achterberg in the key in van Achterberg (2002), but differs by having the head mainly dark brown (much paler in *Pseudomegischus celebensis*), the propodeum regularly scrobiculate (partly rugose), the mesopleuron with large smooth interspaces between medium-sized to small punctures (with larger punctures and narrower interspaces) and the ivory streak of the temple distinct (obsolescent).

#### Description.

Holotype, female, length of body 16.7 mm, and of fore wing 8.9 mm.

*Head*. Antenna with 39 segments; frons coarsely obliquely rugose; three anterior coronal teeth large and acute, both posterior ones arcuate and lamelliform, with two small lobe-shaped carinae on each side in front of both posterior ocelli; behind level of coronal area having four curved, progressively smaller carinae followed by rugose area, rugae rather coarse, posteriorly narrowly reaching occipital carina and widely smooth laterally; temple non-angulate (Fig. 10), punctulate but largely smooth and shiny.

*Mesosoma*. Neck short and robust, transversely rugose, neck at much lower level than middle part of pronotum (Figs 5, 6); middle part of pronotum largely smooth and without a distinct carina posteriorly; propleuron with sparse large punctures, shiny and rather densely setose; mesonotum irregularly foveolate and area between smooth; notauli and median groove distinct; scutellum with some coarse punctures medially, foveolate laterally; axillae coarsely punctate; mesopleuron distinctly convex, convex part foveolate-punctate and covered with long whitish setae, medially convex part of metapleuron rugose and with long whitish setae, anteriorly crenulate and intermediate area smooth; propodeum densely irregularly rugose (Fig. 6).

*Wings*. Fore wing (Fig. 4): vein 1-M 3.0 × as long as vein 1-SR and curved; vein r ends slightly before level of apex of pterostigma; first subdiscal cell robust, 2.9 × as long as its maximum width, vein cu-a entirely pigmented.

*Legs*. Hind coxa robust, without tubercle dorsally and with strong and sparse rugae (Figs 7, 8); hind femur widened, sparsely punctate and with whitish setae ventrally and dark brown setae dorsally, area in between punctures smooth and shiny, ventrally with 2 large acute teeth (the anterior one smaller than posterior one) and several denticles in between (Fig. 7); hind tibia 1.1 × as long as hind femur, basal narrow part of hind tibia about 1.2 × as long as widened part, widened part ventrally distinctly obliquely carinate (Fig. 7); hind basitarsus subparallel-sided, length of hind basitarsus 5.3 × as long as wide medially and 3.6 × as long as second tarsal segment (Fig. 7).

*Metasoma*. First tergite 6.0 × as long as its maximum width (Fig. 12), 1.4 × as long as second tergite, cylindrical, largely smooth except irregular rugae basally and some oblique aciculation after middle of tergite; remainder of tergites smooth and shiny; setose part of ovipositor sheath 1.1 × as long as body and 2.3 × as long as fore wing.

*Colour*. Black or blackish brown; tegula and palpi dark brown; scapus, pedicellus, malar space, mandible and patch near basal quarter of hind tibia partly brown; base of femora and of fore and middle tibiae and tarsi pale yellowish brown; remainder of fore and middle tibiae brown; veins and pterostigma largely dark brown, but base of pterostigma ivory; wing membrane slightly brownish but fore wing darkened near vein r and below parastigma; ovipositor sheath blackish apically (Fig. 1).

*Male*. Similar to female, but in most cases much smaller (Figs 2, 3).

*Variation*. Length of body of ♀ 15–19 mm, of ♂ 8–16 mm; length of fore wing of ♀ 8–11 mm, of ♂ 5–9 mm; antenna of ♀ with 38(1), 39(1), 42(1) or 47(1) segments, of ♂ with 28(2), 29(1), 31(1), 32(2) and 36(2) segments; first metasomal tergite entirely transversely striate or rugose to largely smooth and only basally rugose; hind basitarsus dark brown as hind tibia or brown and paler than hind tibia; length of ovipositor sheath 2.3–2.5 × fore wing.

#### Distribution.

Oriental: China (Jiangxi).

#### Biology.

Reared from stems of *Castanopsis kawakamii* Hay. and *Castanopsis faberi* Hance infested by Cerambycidae and Siricidae.

#### Etymology.

Named after the area of origin, “notios” being Greek for “southern”.

## Supplementary Material

XML Treatment for Pseudomegischus

XML Treatment for Pseudomegischus notiochinensis
